# Management of Pregnancy in a Chilean Patient with Congenital Deficiency of Factor VII and Glanzmann's Thrombasthenia Variant

**DOI:** 10.1155/2014/628386

**Published:** 2014-12-01

**Authors:** Nigel P. Murray, Claudio Garcia, Javier Ilabaca, Nestor Lagos

**Affiliations:** ^1^Department of Hematology, Hospital de Carabineros de Chile, Simon Bolivar 2200, Nuñoa, 7770199 Santiago, Chile; ^2^Faculty of Medicine, University Mayor, Renato Sanchez 4369, Las Condes, 7550224 Santiago, Chile; ^3^Feto-Maternity Unit, Hospital de Carabineros de Chile, Simon Bolivar 2200, Nuñoa, 7770199 Santiago, Chile

## Abstract

Patients with inherited bleeding disorders are rare in obstetric practice but present with prolonged bleeding even after minor invasive procedures. They require a combined approach with obstetric and hematological management of each case, including the neonatal management of a possibly affected fetus. We present the case of a pregnancy in a patient with combined Factor VII deficiency and Glanzmann's thrombasthenia, the successful obstetric and hematological management of the case, and a review of the literature.

## 1. Introduction

Obstetric protocols for the management of patients with inherited bleeding disorders vary regarding the possible need for systemic treatment of haemostatic support, for example, factor concentrates, plasma, and desmopressin, during treatment of patients with inherited bleeding disorders. Obstetric surgical procedures are associated with postoperative bleeding, which is generally self-limiting. However, in patients with an inherited bleeding disorder, relatively minor invasive procedures may precipitate prolonged bleeding. This excessive bleeding is distressing for both patients and clinicians and can delay completion of the procedure, compromise wound healing, and predispose to infection.

We present the case of a 21-year-old pregnant woman with congenital deficiency of Factor VII and Glanzmann's thrombasthenia, a platelet dysfunction disease.

Congenital Factor VII deficiency, first described by Alexander et al. in 1951 [1], is an autosomal recessive condition that produces severe deficiency in the homozygote and moderate deficiency without a bleeding tendency in the heterozygote, with an estimated incidence of 1 in 500,000 in Caucasians [2]. In severe Factor VII deficiency Factor VII, levels cannot reliably determine the clinical manifestations. Patients with Factor VII deficiency are often asymptomatic but may bleed during invasive procedures. Severe bleeding tends to occur in individuals with Factor VII activity levels of 2% or less of normal. Other clinical presentations include spontaneous epistaxis, deep subcutaneous bleeding, genitourinary and gastrointestinal hemorrhages, and hemarthrosis, which are the common manifestations, but can occasionally result in fatal bleeding [3].

Glanzmann's thrombasthenia was first identified in 1918 [4] and described as an inherited disorder characterized by platelets of normal size that failed to spread on a surface and failed to support clot retraction [5]. The defect in platelet aggregation is directly related to a defect in the membrane glycoprotein IIb-IIIA, which is either absent in type I (<5% GPIIb-IIIa) and type II (5–20% GPIIb-IIIa) or variant (based on typical platelet function abnormalities in spite of platelet GPIIb-IIIA concentrations that are 50–100% of normal) [6]. It is an autosomal recessive disease caused by abnormality of the platelet membrane glycoprotein GPIIb-IIIa resulting in absent or decreased platelet aggregation. The clinical course is variable and may be unpredictable in the same patient. Clot retraction is also affected which leads to delayed wound healing. The signs of Glanzmann's thrombasthenia occur early in life and include easy bruising, epistaxis, menorrhagia, postpartum bleeding, and surgical bleeding which can be life-threatening [7, 8]. Heterozygotes with half the normal concentration of platelet GPIIb-IIIa have no abnormalities of platelet function and no clinically significant bleeding [8].

## 2. Clinical Case

A 21-year-old Chilean woman presented to the Department of Obstetrics with a pregnancy of 8 weeks. She had previously been diagnosed at the age of 14 years with congenital deficiency of Factor VII and Glanzmann's thrombasthenia as a result of a family history and evaluation for menorrhagia. Her parents both had a normal prothrombin time and bleeding time as did two brothers and one sister. Her remaining sister had a prolonged prothrombin time, 37% with a Factor VII level of 6% and a bleeding time of 11 minutes and 30 seconds (normal range 2–8 minutes), and had been diagnosed as result of recurrent epistaxis and menorrhagia.

The patient's blood tests revealed a prothrombin time (PT) of 39% (normal range 70–100%) with a Factor VII level of 5% (normal 50–100%), a thromboplastin time of 34 seconds (normal range 23–39 seconds), a platelet count of 213,000/mm³ with normal morphology, and a bleeding time of 9 minutes, 30 seconds (normal range 2–8 minutes). Platelet aggregation studies showed decreased aggregation with epinephrine (15% normal), ADP (17% normal), and collagen (10% normal) and normal aggregation with ristocetin. However, GPIIb-IIIa expression on the platelets as determined by flow cytometry was 48%. This is consistent with the variant type of Glanzmann's thrombasthenia, with reduced aggregation and near normal levels of GPIIb-IIIa expression.

She had not had previous dental extractions or surgery and there was no history of epistaxis, gingival bleeding, or hemorrhagic episodes. Apart from the oral anticontraceptive, she was not on medication.

Pregnancy was uneventful with no episodes of hemorrhage and fetal growth was normal. An elective induction of labor was planned at 39 weeks, with support using fresh frozen plasma (FFP) at a dose of 15 mL/kg to maintain the PT > 50% and platelet transfusions at a dose of 5 units/day to maintain the bleeding time in the normal range. Eight hourly oral tranexamic acid at a dose of 500 mg was planned to start at day −1 to day +20 postpartum. At the end of pregnancy, the prothrombin time of the patient was 46% and the bleeding time was 9 minutes, and FVII levels had increased to 9% but platelet aggregation studies and expression of GPIIb-IIIa were not repeated.

The patient went into labor at 38 weeks and 5 days. During labor, the patient underwent emergency caesarian section owing to a suspected fetal distress. The patient gave birth to a 3.8 kg girl with Apgar scores of 7 and 8; the baby had a normal prothrombin and bleeding time. Surgery was complicated by uterine atony and hemorrhage, which was managed with fluid replacement, hemocomponents, oxytocin, ergomovine, misoprostol, and carbetocin according to standard practice [9]. The patient finally required transmural uterine compression sutures using the B-Lynch method, which resulted in hemostasis.

Table 1 shows the daily FFP and platelet requirements based on the PT and bleeding time. The tests were performed before transfusion of either FFP and/or platelets. As there was a functional response to platelet transfusion with a shortening of the bleeding time, platelet increments after transfusion were not determined. Clinically, there was no significant hemorrhage following caesarian and she was discharged from hospital on day 6.

Outpatient control 10 days after surgery was normal with no signs of hemorrhage; PT was 35% with a bleeding time of 9 minutes, 10 seconds. A second control at 20 days showed a healed wound, but no signs of external hemorrhage, and a pelvic ultrasound showed no collections. The PT and bleeding time were prolonged and in the usual range for the patient. A total of 25 u de platelets and 14 u of FFP were used as profilaxis, and during surgery a further 2 u red cells, 2 u FFP, and 5 u of platelets were transfused.

## 3. Discussion

Factor VII deficiency should be suspected in patients with a prolonged prothrombin time and normal thromboplastin time and the diagnosis confirmed with the Factor VII specific assay. Patients taking oral anticoagulants and/or vitamin K deficiency should be excluded based on the clinical history and in Factor VII deficiency there is failure to improve the prothrombin time with vitamin K. It has been reported that many surgical procedures in patients with deficient Factor VII are well tolerated and may be performed without replacement therapy, such as caesarian section, tonsillectomy, and circumcision [10]. It has been proposed that replacement therapy should be available for use if required. There is no recommended level required for cesarean section and we recommended that an initial level of 50% should be achieved as Factor VII has a short half-life of 2–4 hours. Replacement therapy can be in the form of fresh frozen plasma but carries the risk of volume overload if repeated transfusions are needed. Specific Factor VII concentrates are available, but cost is a prohibitive factor in Chile, whereby a specific dose is given based on weight and degree of Factor VII deficiency. However, unlike hemophilia patients where a specific level of FVIII is associated with bleeding severity this is not true with Factor VII deficiency and replacement therapy is given on a clinical basis, which for the short half-life of Factor VII may need to be frequent. In women heterozygotic for FVII deficiency, there is a significant increase in FVII levels during pregnancy and for this reason postpartum hemorrhages may be lower than expected [11]. In patients with severe FVII deficiency, as in our case, this increase may be limited. Kulkarni et al. [12] reviewed whether or not FVII prophylaxis should be used during delivery. They analyzed 62 women with FVII deficiency and 94 live births between 1953 and 2011. Women undergoing caesarean section were 2.9 times as likely to receive prophylaxis compared with vaginal delivery. Postpartum hemorrhage occurred in 10% of deliveries with prophylaxis compared with 13% of those that did not. The prepregnancy median serum FVII levels of 5.5% were similar between the two groups and the authors concluded that prophylaxis is not necessary. However, they recommended that recombinant FVIIa should be available if needed.

Current guidelines recommend a dose of 90 *μ*g/kg repeated every 2 hours after surgery [13]. A successful delivery by caesarean section using recombinant FVIIa was reported, using a dose of 20 *μ*g/kg 30 minutes before surgery and then every three hours during the 48 hours after surgery. Due to the risk of thrombosis reported with the use of recombinant FVIIa, they used prophylaxis against thrombosis with enoxaparin 4000 UI a day subcutaneously starting 6 hours after surgery for 5 days [14].

The treatment of platelet function disorders such as Glanzmann's thrombasthenia is with platelet transfusions, which again are given on a clinical basis. However, with repeated platelet transfusions, the development of anti-GPIIb/IIIa antibodies and/or anti-HLA antibodies leads to the development of platelet refractoriness. In these patients, recombinant Factor VIIa at a dose of 90 *μ*g/kg appears to be an effective and relatively safe treatment for bleeding and surgical prophylaxis [15]. After FVIIa infusion, there is improved clot structure with increased resistance to proteolysis [14] and thought to act synergistically with platelet activation [16]. In Chile, HLA matched platelets are not available, and therefore the detection of HLA antibodies is not routinely evaluated in these patients. Similarly, the detection of anti-GPIIb-IIIa antibodies is not available, and response to platelet transfusions was based on the bleeding time.

Siddiq et al. [17] reviewed the literature of the management and outcomes of pregnancy in mothers with Glanzmann thrombasthenia; they identified three single center case series and 31 detailed case reports of 40 pregnancies in 35 women. Antenatal bleeding occurred in 50% of cases, which was usually mild and mucocutaneous. Primary postpartum hemorrhage occurred in 34% and secondary postpartum hemorrhage occurred in 24% and was frequently severe. Treatments to prevent and treat postpartum hemorrhage varied, and most women received platelet transfusions, with or without recombinant FVIIa. They also reported that maternal alloimmunization to platelets occurred in 73% of pregnancies.

The use of recombinant FVIIa has been used successfully to treat postpartum hemorrhage, especially in those women in whom platelet transfusions have failed to arrest the hemorrhage, because of antiplatelet antibodies and/or who are refractory to platelet transfusions [18].

In our patient, the logical treatment for combined Factor VII deficiency and Glanzmann thrombasthenia would be recombinant FVII. However, the high cost of recombinant FVIIa is prohibitive in developing countries or where the patient has to assume part or all of the cost. This was the reason for using a combination of FFP and platelet transfusions. We monitored the response to treatment using PT; the use of FVII levels in our hospital is not practical. Although it is possible to measure FVII levels, the samples are sent to another center, with the results being available in five working days, which means that daily monitoring and therapy adjustment are not possible. Therefore, the PT was used as an alternative measure of replacement therapy needs and the bleeding time was used likewise for platelet therapy. The bleeding time is a poor predictor for bleeding during surgery; in patients without a clinical history of abnormal bleeding, the preoperative bleeding time lacks clinical benefit [19]. However, it is useful for assessing platelet function in patients with hemorrhagic disorders [20], and in this case it was used as an indication of therapy response to platelet transfusions.

The patient had been transfused twice previously with a total of 10 units of platelets at the age of 14 years, as there was a good functional response to platelet transfusions as evidenced by the shortening of the bleeding time. We did not evaluate platelet increments after transfusion. The use of HLA matched platelets is not possible in Chile and as such may be an argument for using recombinant FVIIa to prevent the risk of alloimmunization. Similarly, the ongoing surveillance of alloantibody titers is not possible during pregnancy.

Oral eight-hourly tranexamic acid at a dose of 500 mg was used according to the recommendations [13]. General anesthesia was used for the cesarean section as regional anesthetics are contraindicated for the risk of hemorrhage. Uterotonics were used in the third stage of labor as recommended. As recombinant FVIIa was not used in our patient, prophylaxis against thrombosis was not used, the risks of hemorrhage being considered to be greater than thrombosis.

It is important to stress the need for a multidisciplinary approach for managing these patients, and for the newborn infants, who may be affected by thrombocytopenia secondary to maternal transfer of alloantibodies or for inheritance of Glanzmann thrombasthenia and have platelet dysfunction in addition to thrombocytopenia. Given the prevalence of alloimmunization in Glanzmann thrombasthenia and the serious fetal sequelae, fetal thrombocytopenia should be considered in all women with this condition, irrespective of prior platelet transfusions or measurable alloantibodies in the maternal plasma [13]. A rising titre of maternal alloantibody, the development of platelet refractiveness, or a previous pregnancy complicated by fetal thrombocytopenia confers an increased risk of an adverse fetal outcome [18].

Normal pregnancy is associated with hemostatic changes, all contributing to maintaining placental function and preventing excessive bleeding during delivery. During pregnancy, the concentrations of the coagulation factors VII, VIII, IX, X, and XII and von Willebrand factor all rise significantly, accompanied by a relevant increase in the concentration of plasma fibrinogen [21, 22]. Factor VII may increase as much as tenfold during pregnancy [21]. Although in our case the FVII levels only increased marginally to 9%, it may have been sufficient to decrease the risk of hemorrhage. These changes are normally reversed by five weeks after delivery [22].

## 4. Conclusions

We present the successful management of a rare case of combined severe FVII deficiency and Glanzmann thrombasthenia, using FFP and platelet transfusions as prophylaxis, in combination with tranexamic acid and uterotonics in the third stage of labor. Close monitoring of basic tests of hemostasis, the PT, and bleeding time were used to determine the need for replacement therapy.

## Figures and Tables

**Table 1:** Results of daily blood tests and treatment.

	Before surgery	After surgery	6 hours after surgery	Day 2	Day 3	Day 4	Day 5
PT	54%	55%	54%	52%	45%	49%	51%
Bleeding time	6 mins 40 sec	6 mins 15 sec	7 mins	5 mins 45 sec	9 min 30 sec	6 min 50 sec	6 min 30 sec
Treatment	2 u FFP	2 u FFP	2 u FFP	2 u FFP	2 u FFP	2 u FFP	2 u FFP
	5 u plts	5 u plts	5 u plts		5 u plts		5 u plts

PT: prothrombin time; FFP: fresh frozen plasma; plts: platelets.
